# Coupled Investigation of Contact Potential and Microstructure Evolution of Ultra-Thin AlO_x_ for Crystalline Si Passivation

**DOI:** 10.3390/nano11071803

**Published:** 2021-07-12

**Authors:** Zhen Zheng, Junyang An, Ruiling Gong, Yuheng Zeng, Jichun Ye, Linwei Yu, Ileana Florea, Pere Roca i Cabarrocas, Wanghua Chen

**Affiliations:** 1 School of Physical Science and Technology, Ningbo University, Ningbo 315211, China; z13566353862@163.com (Z.Z.); junyang_an@163.com (J.A.); ruiling_gong@163.com (R.G.); 2 Ningbo Institute of Materials Technology and Engineering, Chinese Academy of Sciences, Ningbo 315201, China; yuhengzeng@nimte.ac.cn (Y.Z.); jichun.ye@nimte.ac.cn (J.Y.); 3 National Laboratory of Solid State Microstructures, School of Electronics Science and Engineering/Collaborative Innovation Center of Advanced Microstructures, Nanjing University, Nanjing 210093, China; yulinwei@nju.edu.cn; 4 Laboratory of Physics of Interfaces and Thin Films, CNRS, Ecole Polytechnique, Institut Polytechnique de Paris, 91128 Palaiseau, France; lenuta-ileana.florea@polytechnique.edu (I.F.); pere.roca@polytechnique.edu (P.R.i.C.)

**Keywords:** Kelvin probe force microscopy, c-Si passivation, surface potential, AlO_x_, SiO_x_

## Abstract

In this work, we report the same trends for the contact potential difference measured by Kelvin probe force microscopy and the effective carrier lifetime on crystalline silicon (c-Si) wafers passivated by AlO_x_ layers of different thicknesses and submitted to annealing under various conditions. The changes in contact potential difference values and in the effective carrier lifetimes of the wafers are discussed in view of structural changes of the c-Si/SiO_2_/AlO_x_ interface thanks to high resolution transmission electron microscopy. Indeed, we observed the presence of a crystalline silicon oxide interfacial layer in as-deposited (200 °C) AlO_x_, and a phase transformation from crystalline to amorphous silicon oxide when they were annealed in vacuum at 300 °C.

## 1. Introduction

Dielectrics are widely applied for the passivation of crystalline Si (c-Si) wafers used in c-Si solar cells because they can provide both chemical and field effect passivation. Due to the parasitic shunt formed in the case of an inverted surface [1], dielectrics with negative fixed charge (*Q_f_*), such as Aluminum oxide (AlO_x_), are favorable to p-type c-Si, while dielectrics having a positive *Q_f_*, such as amorphous hydrogenated silicon nitride (a-SiN_x_:H), are better suited to n-type material. Different techniques have been developed to grow AlO_x_ layers: (i) sputtering, which requires an extrinsic hydrogenation step to obtain a good passivation [2]; (ii) atomic layer deposition (ALD) [3,4,5,6,7]; (iii) oxidation of Al with the help of other oxide materials, such as TiO_2_ [8]; (iv) inline plasma-enhanced chemical vapor deposition [9], etc. Among these techniques, ALD can provide extremely good passivation quality. The advantage of ALD is its precise control of the thickness at atomic level with perfect uniformity and conformality. Two main types of ALD have been developed including plasma-enhanced ALD (PEALD) and thermal ALD. By taking advantage of the negative fixed charge density of AlO_x_ itself, a passivation with surface recombination velocity (SRV) close to 10 cm/s has been achieved for p-type silicon [10]. Two main approaches have been developed to improve the passivation quality provided by AlO_x_ including thermal annealing [11,12,13,14] and light-soaking [15,16,17].

The passivation quality mainly depends on the interfacial states or defects between the c-Si substrate and the AlO_x_ layer. Therefore, studying the c-Si/AlO_x_ interface is an important issue. For example, investigation of the interfacial composition with the help of transmission electron microscopy (TEM) has revealed the existence of a mixed SiO_x_ layer [18,19,20]. A high *Q_f_* density, as measured by corona charging experiments, provides a very strong field effect passivation [21]. Second harmonic generation (SHG) measurements have indicated a thickness independent negative *Q_f_* suggesting that the charge must be located at the SiO_x_/AlO_x_ interface [22], which results from the local reconstruction of the interfacial SiO_x_ layer after annealing in Ar ambient at 525 °C for 15 min [23]. Such changes can affect the work function of the material, which depends on its doping level and the presence of surface dipoles and surface states, which can be measured by Kelvin probe [24,25]. Therefore, AlO_x_, layers have been widely studied by Kelvin probe. To cite a few examples: (i) the determination of the surface potential uniformity of AlO_x_ [26], (ii) imaging of the charge in AlO_x_ gate oxides [27], (iii) estimation of the total charge density in metal/AlO_x_/SiO_2_/Si structures [28], and (iv) study of the surface potential difference between AlO_x_-coated graphene and AlO_x_-coated Cu [29]. In this work, we used thermal ALD to deposit AlO_x_ layers. To investigate the properties of AlO_x_ under different conditions including as-deposited state and after thermal treatments, the contact potential difference (*CPD*) of AlO_x_ layers was measured with the help of Kelvin probe force microscopy (KPFM). High resolution TEM (HRTEM) was used to characterize the evolution of the microstructure of AlO_x_ layers under different thermal treatments.

## 2. Experimental Procedure

AlO_x_ layers with three different thicknesses (1.5 nm, 5 nm and 10 nm) were deposited using thermal ALD technique (Savannah Ultratech, Cambridge, UK). The thickness of AlO_x_ is controlled by the number of cycles. Double side polished Si wafers (FZ, (100) n-type, 4-inch diameter, 250 μm, 1–10 Ω·cm) were used as substrates for a symmetrical deposition. The same deposition temperature of 200 °C was used for all AlO_x_ passivation layers. The detailed ALD deposition conditions are presented in Table 1. In order to remove the native oxide, the c-Si wafers were dipped into HF (5%) for 30 s prior to AlO_x_ deposition. KPFM (Asylum research, Oxford instruments, Buckinghamshire, UK) was used to investigate the AlO_x_ passivated samples. Conductive atomic force microscopy (AFM) tips (AC240TM from Olympus, Tokyo, Japan) were used, which have a doped Si cantilever and a Pt coating. The wavelength in the KPFM system is 850 nm. The laser spot size is around 3 × 9 µm, which is smaller than the cantilever. Moreover, the laser spot is well positioned away from the cantilever edge to minimize the parasitic illumination from AFM laser. The KPFM measurements in this work were performed under humidity below 30%, since Sugimura et al. report that low humidity can allow a good potential contrast [30]. The measurements were performed with a planar configuration of the sample since the ultra-thin thickness of AlO_x_ layers will cause interpretation difficulties when investigated in cross-section configuration. A diamond pen was used to scratch the sample surface in order to create a sharp interface between AlO_x_ and the c-Si substrate. Afterwards, an ultrasonic treatment of samples in deionized water was applied for 60 min in order to remove the scratch-produced Si flakes, since too much surface roughness can cause measurement artifacts, difficulty of data interpretation and breaking of tips.

TEM analyses of different AlO_x_ deposited layers required a lamella preparation. Thus, cross-section lamellas were prepared using a standard lift-out procedure within a Focus Ion Beam dual beam microscope (FIB, FEI-Scios DualBeam, Thermo Fisher Scientific, Waltham, MA, USA). Transmission Electron Microscopy (TEM) analyses were performed on 2 different Titan Themis transmission electron microscopes operating at 300 kV and 200 kV accelerating voltage. For the TEM observation, we used the low dose mode of the electron microscope and a 4k/4k direct detection electron (DDE) camera. The DDE camera is a very sensitive camera which uses very low dose (max 25 e^–^/Å^2^) for the image acquisition. In our case, the max dose used with the TEM operating at 300 kV was 10 e^–^/Å^2^. For the chemical analyses we used a Titan-Themis operating at 200 kV equipped with a Cs probe corrector and a SuperX detector that allows chemical analyses of light and heavy elements with a spatial resolution within picometer range. The experimental conditions were set so that the total current within the probe used for the chemical analysis was about 85 pA. As elements of interest, we chose silicon with Kα = 1.74 keV ionization edge, oxygen with K_α_ = 0.523 keV, and aluminum with K_α_ = 1.48 keV ionization edge. Carbon and platinum protective layers were deposited on AlO_x_ layer prior to the FIB milling process in order to prevent the Ga ion implantation during the milling process.

## 3. Results and Discussion

**Figure 1:**
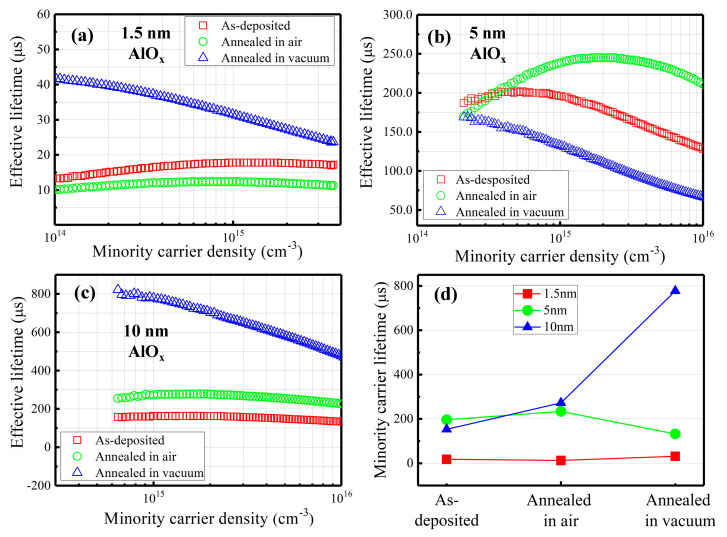
Minority carrier lifetime as a function of the minority carrier density for symmetrical deposition of AlO_x_ with three different thicknesses: (**a**) 1.5 nm, (**b**) 5 nm, (**c**) 10 nm. (**d**) Summary of effective carrier lifetime upon different thermal treatments.

The effective minority carrier lifetime of our samples was measured by quasi steady-state photoconductance (QSSPC, Sinton Instruments, Boulder, CO, USA). The results are presented in Figure 1, where AlO_x_ passivation layers with three different thicknesses (1.5 nm, 5 nm and 10 nm) are shown. Three states of the samples were characterized, including as-deposited, annealed in air and annealed in vacuum (0.1 Pa), separately. The annealing temperature and annealing time are kept the same for all these treatments at 300 °C for 15 min. Note that the effective carrier lifetime was recorded at the injection level of 10^15^ cm^−3^. The corresponding lifetime values are summarized in Figure 1d. The as-deposited sample with 1.5 nm AlO_x_ has a lifetime of 17 µs and it decreases to 12 µs after annealing in air. Its value increases to 31 µs after annealing in vacuum. For 5 nm AlO_x_ passivated sample (Figure 1b), the as-deposited lifetime (200 µs) increases to 233 µs but decreases to 132 µs when annealed in air and vacuum, respectively. For 10 nm of AlO_x_, their lifetime value increases from 152 µs to 272 µs then reaches 777 µs once the as-deposited sample experiences annealing in air and annealing in vacuum, respectively. A conclusion can be made that AlO_x_ passivation with different layer thicknesses exhibits different trends even if they were submitted to the same thermal annealing, indicating that the layer properties and their evolution depend on AlO_x_ layer thickness.

In order to investigate the influence of thermal treatment on passivation quality of AlO_x_ layers, AFM and KPFM were used, where AFM provides information on the morphology and KPFM provides *CPD* values. The corresponding AFM and KPFM mappings of c-Si/AlO_x_ samples are presented in Table 2. The diamond-introduced scratching channel can be clearly observed with some bumps at the scratching edge. A clean c-Si surface without the flakes has been obtained thanks to the ultrasonic treatment.

**Figure 2:**
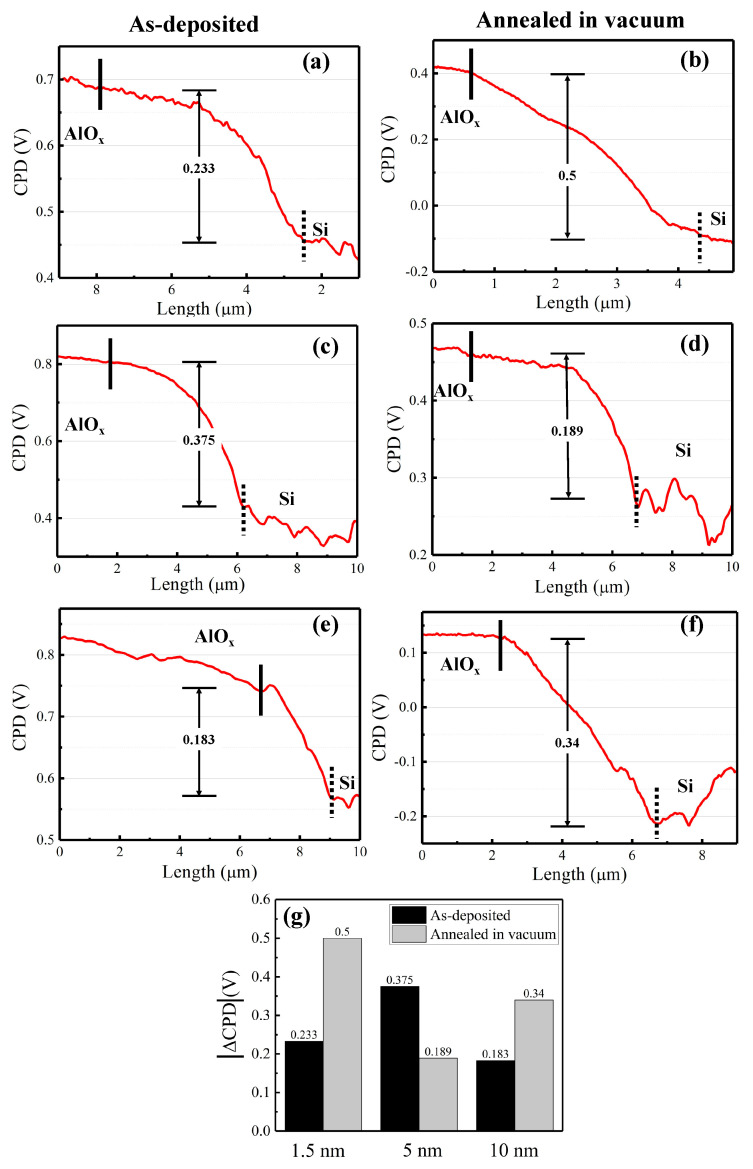
KPFM characterization of six different AlO_x_ passivation samples after annealing in vacuum (right side) as compared to their as-deposited state (left side). (**a**,**b**) 1.5 nm AlO_x_. (**c**,**d**) 5 nm AlO_x_. (**e**,**f**) 10 nm AlO_x_. (**g**) Summary of |Δ *CPD*| values for 1.5, 5 and 10 nm AlO_x_.

In order to have quantitative information from AFM and KPFM, a profile across the scratching channel between AlO_x_ and c-Si substrate is made, marked as red lines in Table 2. Note that both AFM and KPFM profiles were collected from the same position for the same sample. The contact potential difference (*CPD*) between the tip and sample surface can be defined as follows:

(1) $$CPD=\frac{\varphi_{t}-\varphi_{s}}{e}$$

where, $\varphi_{t}$ and $\varphi_{s}$ are the work function of the conductive AFM tip and sample, respectively, and *e* is the elementary charge. The corresponding *CPD* profiles are presented in Figure 2. The *CPD* corresponding to the edge of the flat zone (marked as the solid line) can be defined as the *CPD* of c-Si/AlO_x_ layer stacks. The *CPD* of c-Si substrate surface is defined as the value of the first turning point of the *CPD* curve, which is marked by the black dotted line. The *CPD* difference Δ *CPD* between AlO_x_ and c-Si can be considered as the absolute *CPD* of interfacial layer, which can be defined as follows:

(2) $$\left|\Delta \;CPD\right|=\varphi_{AlOx}-\varphi_{Si}$$

The *CPD* values can be measured as 0.233 V (0.5 V), 0. 375 V (0. 189 V), and 0.183 V (0.340 V) for 1.5, 5 and 10 nm AlO_x_ in the as-deposited state presented by white columns (annealed in vacuum presented by grey columns), respectively, as summarized in Figure 2g. Therefore, we can observe that the *CPD* of c-Si/AlO_x_ for 1.5 and 10 nm sample increases after annealing in vacuum, while it decreases for 5 nm AlO_x_. Based on Equation (2), we can calculate that the work function of a given layer increases with the increase of its Δ *CPD*. Then, we can infer that, for 1.5 and 10 nm AlO_x_, the work function of c-Si/AlO_x_ increases, while for 5 nm, it decreases. Comparing with the corresponding minority carrier lifetimes in Figure 1d, we can conclude that the changes in the work function follow the same trends as the effective minority carrier lifetime.

**Figure 3:**
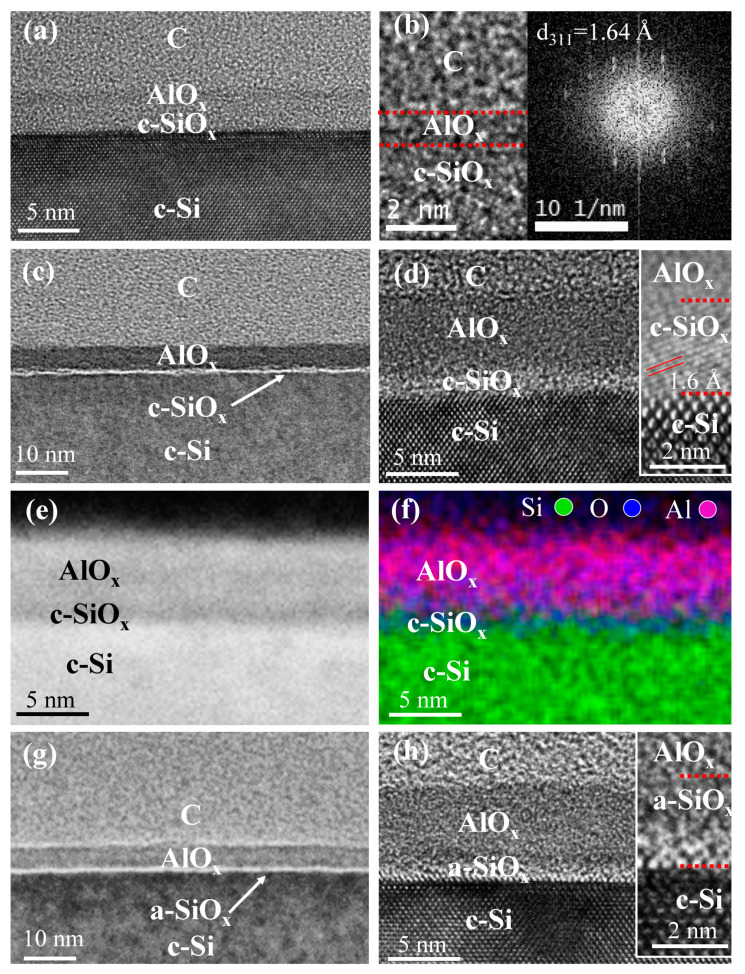
(**a**) HRTEM image of as-deposited 1.5 nm AlO_x_ layer, with the enlarged view in (**b**). Inset showing the Fourier transform. (**c**) Low magnification TEM image of as-deposited 5 nm AlO_x_ layer with HRTEM image shown in (**d**). (**e**) STEM-HAADF image of as-deposited 5 nm AlO_x_ layer. (**f**) STEM-HAADF EDX mapping of as-deposited 5 nm AlO_x_ layer. (**g**) Low magnification TEM image of 5 nm AlO_x_ layer annealed in vacuum and the corresponding HRTEM image in (**h**). Insets of the figure (**b**,**d**,**h**) show the corresponding HRTEM images, evidencing the crystalline/amorphous character of different layers. Red dashed lines are provided as a guide to the eye for different layers.

To understand the work function evolution for AlO_x_ layers, TEM was used to study their microstructure under different thermal treatments. The characterization results are shown in Figure 3. A HRTEM image of as-deposited 1.5 nm AlO_x_ layer is shown in Figure 3a with a zoom of interface shown in Figure 3b, where a partially crystallized amorphous SiO_x_ (a-SiO_x_) phase can be observed. The inset of Figure 3b shows the corresponding Fourier transform. The inter plane distance can be calculated to be 1.64 Å. The low magnification and HRTEM images for 5 nm AlO_x_ are presented in Figure 3c,d, respectively. Inset of Figure 3d shows the corresponding HRTEM image, where the interfacial SiO_x_ layer reveals again a crystalline structure with inter plane distance of 1.6 Å. The oxidation of Si substrate could be due to the interfacial oxygen diffusion during AlO_x_ deposition. The chemical distribution of different elements of the SiO_x_ layer is also studied by scanning transmission electron microscopy-high angle annular dark-field (STEM-HAADF) and energy dispersive X-ray (EDX) spectroscopy. The STEM-HAADF image is shown in Figure 3e. A closer analysis of STEM-EDX mapping allowed the identification of the multilayer structure as shown in Figure 3d, with the clear crystalline SiO_x_ (c-SiO_x_) layer at the interface between the c-Si wafer and AlO_x_. The total crystallization of interfacial SiO_x_ occurs during longer deposition time (thicker AlO_x_ layer). Surprisingly, when the AlO_x_ layer is annealed in vacuum, the amorphization of the c-SiO_x_ layer occurs as shown in Figure 3g,h, which correspond to low and high magnification of TEM, respectively, with its HRTEM image shown in the inset.

The evolution of the surface passivation provided by the AlOx layers submitted to different annealing conditions can be discussed based on the presence of hydrogen in AlO_x_ layers [31,32]. The difference in ambient conditions can affect the diffusion of hydrogen, which can be more important for the sample annealed in vacuum compared to that of the sample annealed in air. Concerning the 1.5 nm AlO_x_ passivation layer, in the case of annealing in air, the SiO_x_ layer turns out to be more crystallized, probably due to exothermic reactions of H confined at the interface. In the case of 5 nm AlO_x_ passivation, when annealed in air, a relatively thicker AlO_x_ layer could effectively prevent hydrogen atoms from escaping, and eventually keep them at the c-Si/AlO_x_ interface to form a hydrogen-saturated chemical passivation. However, when annealing in vacuum, the hydrogen in the passivation layer can be pumped away; hence, it cannot effectively saturate the dangling bonds. Therefore, the passivation quality after annealing in vacuum is even lower than that of the as-deposited sample because of dehydroxylation at the AlO_x_ layer. As compared to 5 nm AlO_x_ passivation, more Al-OH bonds are formed during the deposition of the 10 nm AlO_x_ layer. Thus, the thicker AlO_x_ layer can prevent the dehydroxylation even when annealed in vacuum. When it is annealed in air, a slightly better passivation quality can be obtained because of the inhibition of dehydroxylation due to the ambient air conditions. However, when the sample is annealed in vacuum, dehydroxylation occurs more easily. As a consequence, more H atoms can be released from Al-OH bonds. Therefore, a better passivation quality can be obtained when 10 nm AlO_x_ layer is annealed in vacuum. 

From the above HRTEM results, we can conclude that for the sample annealed in vacuum, the SiO_x_ interfacial layer exhibits an amorphous structure, while its thickness is maintained at the same value as for the as-deposited state. It has been reported that a thin amorphous AlO_x_ is more stable than a crystalline one due to the surface and interface defects [33]. Therefore, one can expect that the amorphous state of the interfacial SiO_x_ is favored as compared to its crystalline state when annealed at 300 °C for 15 min. The decrease of the work function upon annealing for the 1.5 nm AlO_x_ layer contrasts with the 5 and 10 nm AlO_x_ layers and can be discussed as follows. The work function values for c-Si and a-Si are 4.85 eV and 4.66 eV, respectively. Accordingly, we suggest a tendency that the work function of SiO_x_ decreased from crystalline to amorphous. Therefore, a higher work function of c-SiO_x_ is expected as compared to a-SiO_x_. Based on the HRTEM images in Figure 3d,h, the interfacial SiOx changed from crystalline to amorphous state after annealing; therefore, the corresponding work function decreases. Literature reported that in a nonzero net charge sample including *Q_f_*, a higher work function can be achieved compared to zero net charge sample [34]. Therefore, we can assume that AlO_x_ with higher work function has larger *Q_f_*. For 1.5 and 10 nm AlO_x_, their work functions increase after annealing in vacuum resulting from an increase of *Q_f_* and, therefore, leading to an improvement of the passivation quality. On the contrary, a decrease of work function due to a lower *Q_f_* will cause a degradation of the passivation quality in the case of 5 nm AlO_x_. This is consistent with minority carrier lifetime results as presented in Figure 1.

## 4. Conclusions

In summary, the influence of thermal treatments on AlO_x_ with three thicknesses (1.5 nm, 5 nm and 10 nm) was investigated by KPFM and HRTEM. We have found that AlO_x_ passivation with different layer thicknesses exhibits different trends even if they were exposed to the same thermal annealing. The passivation quality increases when AlO_x_ was annealed in vacuum for 1.5 and 10 nm, while it decreases for the 5 nm sample. From KPFM measurements of samples after annealing in vacuum, an increase of work function is observed in the case of 1.5 and 10 nm AlO_x_ layers, while a decrease of work function is obtained for the 5 nm AlO_x_ layer, which has been related to the amorphization of the interfacial crystalline SiO_x_ layer as evidenced by HRTEM.

## Figures and Tables

**Table 1:** ALD cycle used in the work. Deposition chamber is flushed by 20 SCCM of N_2_. (SCCM: standard cubic centimeters per minute).

	TMA	Purge	H_2_O	Purge
Time (s)	0.015	3	0.015	3

**Table 2:** AFM (morphology) and KPFM (*CPD*) mapping of six AlO_x_ samples: as-deposited and annealed in vacuum for three different AlO_x_ thicknesses.

Samples	As-Deposited		Annealed in Vacuum	
	Morphology	*CPD*	Morphology	*CPD*
1.5 nm	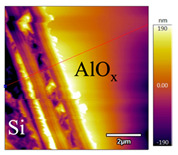	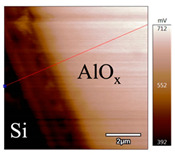	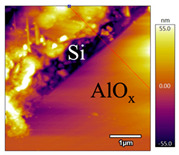	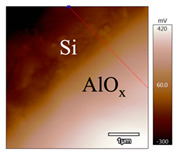
5 nm	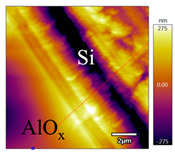	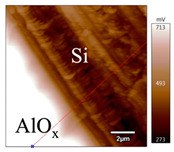	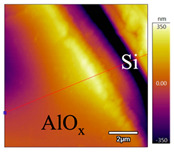	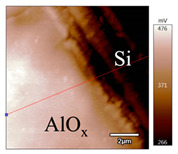
10 nm	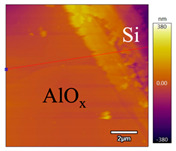	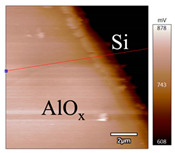	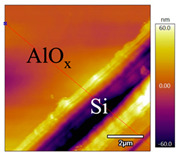	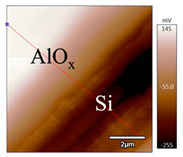

## Data Availability

Data are contained within the article.
